# Optimizing Analytical Thresholds for Low-Template DNA Analysis: Insights from Multi-Laboratory Negative Controls

**DOI:** 10.3390/genes15010117

**Published:** 2024-01-18

**Authors:** Dezhi Chen, Mengyu Tan, Jiaming Xue, Mengna Wu, Jinlong Song, Qiushuo Wu, Guihong Liu, Yazi Zheng, Yuanyuan Xiao, Meili Lv, Miao Liao, Shengqiu Qu, Weibo Liang

**Affiliations:** 1 Department of Forensic Genetics, West China School of Basic Medical Sciences and Forensic Medicine, Sichuan University, Chengdu 610041, China; chendz2023@163.com (D.C.); tanmengyu1996@gmail.com (M.T.);; 2 Department of Immunology, West China School of Basic Medical Sciences and Forensic Medicine, Sichuan University, Chengdu 610041, China; 3 West China Forensics Center, Sichuan University, No. 16, Section 3, Renmin South Road, Wuhou District, Chengdu 610041, China

**Keywords:** analytical threshold, forensic genetic analysis, low-template DNA, short tandem repeats, capillary electrophoresis, negative control

## Abstract

When analyzing challenging samples, such as low-template DNA, analysts aim to maximize information while minimizing noise, often by adjusting the analytical threshold (AT) for optimal results. A potential approach involves calculating the AT based on the baseline signal distribution in electrophoresis results. This study investigates the impact of reagent kits, testing quarters, environmental conditions, and amplification cycles on baseline signals using historical records and experimental data on low-template DNA. Variations in these aspects contribute to differences in baseline signal patterns. Analysts should remain vigilant regarding routine instrument maintenance and reagent replacement, as these may affect baseline signals. Prompt analysis of baseline status and tailored adjustments to ATs under specific laboratory conditions are advised. A comparative analysis of published methods for calculating the optimal AT from a negative signal distribution highlighted the efficiency of utilizing baseline signals to enhance forensic genetic analysis, with the exception of extremely low-template samples and high-amplification cycles. Moreover, a user-friendly program for real-time analysis was developed, enabling prompt adjustments to ATs based on negative control profiles. In conclusion, this study provides insights into baseline signals, aiming to enhance genetic analysis accuracy across diverse laboratories. Practical recommendations are offered for optimizing ATs in forensic DNA analysis.

## 1. Introduction

International law enforcement and justice entities have reached a consensus that DNA analysis is the “gold standard” in forensic investigations. Short tandem repeats (STRs), characterized by their widespread distribution and high genetic polymorphism within populations, have emerged as pivotal genetic markers for investigative procedures. Common crime scene traces such as cigarette butts, hair, and fingerprints often present challenges because of their low-template DNA, which is susceptible to random effects during polymerase chain reaction (PCR) amplification, including allelic imbalance and allele dropout [1]. These factors add complexity to subsequent STR profile analyses.

To address allele dropout (Type II error), various sensitivity-enhancing methods have been explored, such as increasing PCR cycles, reducing PCR volume, using nested PCR, enhancing fluorescent dye signals, extending injection times, and employing higher-purity formamide during sample preparation for capillary electrophoresis [2,3,4]. However, the heightened sensitivity increases the risk of mislabeling non-allelic signals (Type I error), which can arise from PCR products (e.g., stutter, non-template-dependent nucleotide addition, and non-specific amplification products) or instrumental artifacts (e.g., spikes, raised baselines, and incomplete spectral separation resulting in pull-up or bleed-through) [5]. Many labs currently adhere to the recommended analytical threshold (AT) provided by amplification kit manufacturers when analyzing forensic samples. This conservative approach aims to minimize the impact of background noise and PCR artifacts. However, for low-template samples, conservative ATs cannot reliably differentiate the target DNA signal from noise [6]. Additionally, in cases of limited sample quantity, it is impractical to further increase the detection sensitivity and conduct retests. The SWGDAM Interpretation Guidelines emphasize that “an AT defines the minimum height requirement at and above which detected peaks can be reliably distinguished from background noise. Peaks above AT are generally not considered noise, and are either artifacts or true alleles” [5]. Therefore, to ensure optimal signal processing parameters during DNA analysis, it may be advisable to select an AT that minimizes both Type I and Type II errors.

In practice, various forensic DNA analysis laboratories employ different methods to determine the threshold for analysis. Gilder et al. [7] proposed a method endorsed by the IUPAC, based on Kaiser’s suggestion [8,9]. In this approach, a threshold is established by analyzing the baseline noise to ensure that signals arising from random fluctuations are not erroneously labeled as true alleles. Marciano et al. [10] described a dynamic locus and sample-specific AT based on the mean and standard deviation of noise in regions flanking a locus within an individual sample. This system achieved 97.2% accuracy in allele detection, representing an 11.4% increase over the lowest static threshold (50 RFU). Additionally, some methods rely on the relationship between the RFU signal and DNA input into PCR, originating from the field of chemical analysis and later applied to DNA analysis [11,12,13,14]. Several previous studies compared various methods for determining the optimal AT. Rakay et al. [14] separately tested ATs derived from negatives, the relationship between RFU signals and DNA input, and commonly employed ATs to compare their impact on both Type I and Type II errors. They suggested that, for samples amplified with less than 0.5 ng DNA, applying ATs derived from baseline analysis of negatives can reduce the probability of allele dropout by a factor of 100 without significantly increasing the probability of erroneous noise detection. Bregu et al. [15] also outlined and compared four different methods that rely on the analysis of baseline noise from a number of negatives to calculate ATs. They found that variations in the procedural conditions could affect the baseline noise associated with genetic analysis, ultimately influencing the determination of the analysis threshold in laboratories. They also recommended the use of ATs derived from negative samples with lower DNA levels. However, despite the abundance of methods for calculating ATs based on negative signals, there is still no clear consensus on the preferred method for practical casework. Law enforcement agencies face a challenge in the absence of a scientific guide and framework for adjusting ATs, particularly for low-template samples.

This study analyzed the status and distribution of baseline noise across multiple laboratories over three years, considering reagent kits, testing quarters, laboratory conditions, and amplification cycle numbers, using a large number of negative control profiles. The objective of this study was to explore the need for each laboratory to establish an optimal AT. In this study, we utilized established methods, relying on amplification negatives, to determine ATs for analyzing low-template DNA profiles. The goal was to compare error rates across laboratories. The overall objective of this study was to establish a universally applicable AT calculation practice model for scientific and efficient genetic analysis, providing guidance and references for laboratory personnel in diverse settings.

## 2. Materials and Methods

### 2.1. Collection of Historical and Experimental Data

In total, 929 negative control samples were collected from six laboratories (LAB_a–f) between 2019 and 2022. The amplification kits used included the AGCU EX22 kit (Applied ScienTech, Wuxi, China), PowerPlex 21 kit (Promega, Madison, WI, USA), and VeriFiler™ Plus kit (Thermo Fisher Scientific, Waltham, MA, USA). Amplified products were analyzed using an ABI 3500 Genetic Analyzer (Applied Biosystems, Foster City, CA, USA). 

Additionally, the experiments on low-template DNA samples were conducted by seven laboratories (LAB_a–e, g and h) using the VeriFiler™ Plus kit. All experiments were consistently performed by the same experimenter. Female control DNA 9947A (OriGene, Rockville, MD, USA) was diluted to three concentrations: 31.25 pg/µL, 15.625 pg/µL, and 7.8125 pg/µL. Each PCR reaction used 1 µL of DNA, with a total volume of 10 µL, following the routine protocol for the cases. Three PCR replicates were conducted for each concentration of 9947A and negative control for 27, 29, and 31 cycles. The amplified products were separated via capillary electrophoresis using an ABI 3500 Genetic Analyzer (Applied Biosystems, Foster City, CA, USA), with three replicates for each amplified product. The research protocol was reviewed and approved by the Ethics Committee at the Institute of Forensic Medicine, Sichuan University (No. KS2022770) (4 March 2022).

### 2.2. Analysis of Large-Scale Negative Control Samples

The negative control results from historical records and low-template DNA experiment were analyzed using GeneMapper ID-X. First, the empirical AT commonly used in each laboratory was used to analyze all negative samples. Negative samples with peak heights above this threshold were excluded. Subsequently, an AT of 1 RFU (with the threshold of the internal lane standard set at 175 RFU) was used to analyze the remaining negative samples. The data from the “Sizing Table” in GeneMapper ID-X for each dye were exported, containing the details of each signal above the AT, such as marker, allele, size, height, area, and data point. An in-house Python script was used to filter signals outside the read region recommended by the manufacturer. All signals within 2 bases of the internal lane standard were removed to avoid the influence of pull-up [15]. Negative samples from each laboratory were then grouped into quarters at three-month intervals. The signal number and height distribution for each dye across the quarters and laboratories were analyzed.

### 2.3. Study on ATs

The low-template DNA results were analyzed using GeneMapper ID-X, with a minimum AT set at 1 RFU for each non-internal standard dye. Signal data from the “Sizing Table” for each dye were exported and processed using a Python script. This script initially screened the peaks occurring at the locus positions. Subsequently, to eliminate pull-up peaks, it excluded signals meeting specific criteria: sharing the same position (±0.3 bases) as an allelic peak in another dye, and with a peak height of 5% or less compared to that of the allelic peak [16]. The AT for each dye varied from 1 to 200 RFU. The number of allelic dropouts and non-allelic peaks at each AT were statistically analyzed with the reference control DNA 9947A with 1 ng input.

In addition, low-template control DNA samples were analyzed using GeneMapper ID-X with six distinct groups of ATs. These ATs included one conventional threshold (denoted *AT_ori_*) and five thresholds calculated using previously published methods [7,8,9,12,15,17,18]. The *AT_ori_* set uses a specific threshold value of 175 RFU for each dye. The other five methods were developed based on the analysis of signals from negative samples in low-template DNA experiments. 

*AT*_1_: *AT*_1_ was calculated using the following equation:

(1) $${AT}_{1}=Y_{n}+k\cdot s_{Y,n}$$

where *Y_n_* is the mean of the negative signals, *s_Y,n_* is the standard deviation of the negative signals, and *k* is a constant that depends on the desired confidence level. In accordance with insights from the preceding literature on the choice of *k* [7,8,12,13,14], this study opted to set *k* equal to three.

*AT*_2_: The following equation was used to determine *AT*_2_:

(2) $${AT}_{2}=Y_{n}+t_{\alpha ,\upsilon }\cdot \frac{s_{Y,n}}{\sqrt{n_{n}}}$$

where *Y_n_* and *s_Y,n_* are the mean and standard deviation of the negative signals, respectively, *t_α,υ_* is the one-sided critical value from the t-distribution for a given confidence interval, and *n_n_* is the number of negative samples. 

*AT*_3_: *AT*_3_ was computed using the following equation:

(3) $${AT}_{3}=Y_{n}+t_{\alpha ,\upsilon }\cdot {\left(1+\frac{1}{n_{n}}\right)}^{\frac{1}{2}}\cdot s_{Y,n}$$

where the parameters are defined as in the equation for *AT*_2_, and $\left(1+\frac{1}{n_{n}}\right)$ expresses the correction for the uncertainty of the true and calculated mean negative signal.

*AT*_4_: *AT*_4_ indicates the level of background noise in a signal. This was calculated by determining the value that separated 99% of the negative signals from the rest. 

*AT*_5_: The following equation was used to determine *AT*_5_:

(4) $${AT}_{5}=e^{\upsilon +k\tau }$$

The calculation of *AT*_5_ was based on the assumption that a negative signal follows a lognormal distribution. Consequently, the natural logarithm (log base *e*) of the negative signal is assumed to follow a normal distribution, with a mean denoted as *υ* and a variance represented by *τ*. The specific value of factor *k* depends on the chosen confidence level used to estimate the noise. The value of *k* was set to three. 

The exported “Sizing Table” at each AT group underwent further analysis. The number of allelic dropouts and non-allelic peaks in the low-template DNA samples under different DNA inputs and amplification cycles were counted and analyzed. Receiver operating characteristic (ROC) curves were plotted for the six AT groups based on the true-positive and false-positive results. ROC analysis was used to determine the optimal method for determining ATs in different laboratories under different conditions.

### 2.4. Building of the Executable Program NegaProcess 

Utilizing PyInstaller-6.1.0 (http://www.pyinstaller.org/, accessed on 20 October 2023), the signal analysis script for negative samples and the calculation script for five AT calculation methods based on negative signals were compiled into an executable program named NegaProcess. This versatile program empowers researchers to effortlessly load any quantity of negative control samples, assess the baseline conditions of their laboratories, and scientifically adjust the AT to enhance sample analysis. Importantly, it does not demand programming or statistical expertise, allowing users to save time that would otherwise be spent on learning analysis procedures.

## 3. Results

### 3.1. Characteristics of Negative Signals

Negative samples collected over time were categorized into quarterly divisions, with each quarter comprising three months. The yearly timeframe was divided into four quarters, denoted as S1–4. Table 1 provides an overview of the distribution of the available negative samples collected from various laboratories. Distinct superscript numbers indicate the different STR kits used.

**Figure 1:**
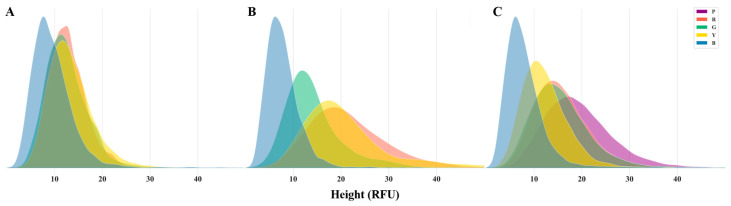
The comparison of signal height distribution of various dyes obtained from negative samples using the AGCU EX22 (**A**), PowerPlex^®^ 21 System (**B**), and VeriFiler™ Plus (**C**). Distributions distinguished by various colors correspond to different dyes.

Analysis of the three PCR kits revealed comparable numbers of signals for each dye in each kit, with no significant differences observed (Table S1). The graphical representation displays a right-skewed normal distribution of signal heights for each dye (Figure 1). Subsequent fitting and validation confirmed the lognormal distribution of the signal heights, which was consistent with prior research findings [15]. Notably, distinct and consistent disparities were noted in the signal height distributions among the dyes in the three kits. The blue dye consistently exhibited the smallest average signal height across all the kits. For the AGCU EX22 kit, no significant differences were observed in the signal height distributions of other dyes. In the PowerPlex^®^ 21 System, a significantly higher number of signals were clustered around the average signal height in the blue dye than in the other dyes. The green dye displayed the second-highest average signal height, with no significant disparities observed in the signal height distribution between the yellow and red dyes. In the VeriFiler™ Plus kit, signals in the blue dye also concentrated around the average height, while the yellow dye exhibited the second-highest average signal height. Notably, the purple dye showed the largest average height, characterized by a broader and shorter distribution in the graphical representation compared to the other dyes. These findings indicate that specific patterns emerged in the variations among the different dyes in terms of negative signals and signal heights. The analysis of common samples may have been influenced to varying extents by the detection of genuine allelic peaks. Consequently, it is necessary to establish a distinct AT tailored to the unique baseline characteristics of each dye to minimize the interference from the baseline signals introduced by the equipment and reagents.

#### 3.1.1. Comparative Analysis of Negative Signal Quantities and Heights across Different Quarters

**Figure 2:**
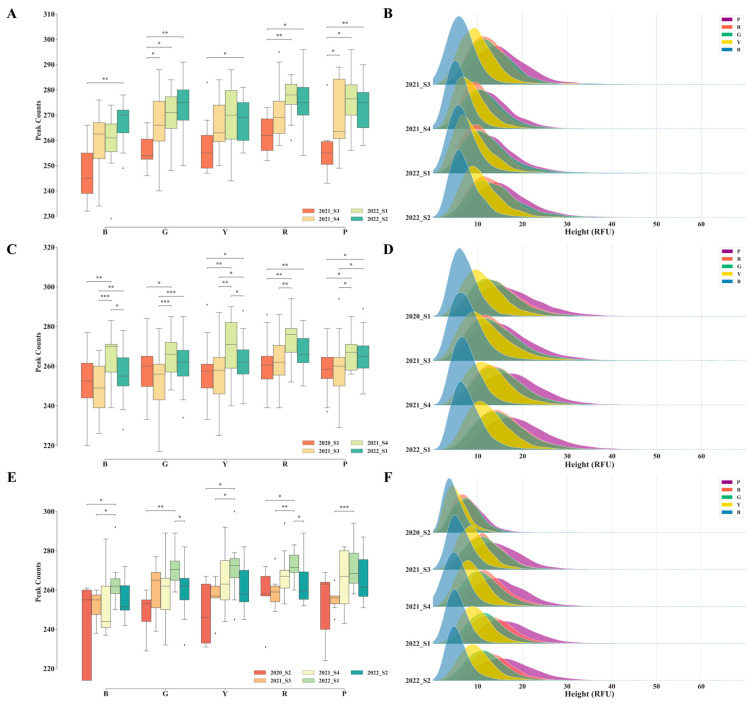
The signal quantities and height distributions in negative samples from three laboratories across different quarters, utilizing the VeriFiler™ Plus kit. Sub-figures (**A**,**C**,**E**) display the signal quantities for LAB_b, LAB_c, and LAB_e, respectively, while sub-figures (**B**,**D**,**F**) showcase the corresponding signal height distributions. The outliers are represented by diamonds. Statistical significance is indicated by symbols: * denotes 0.01 < *p* ≤ 0.05, ** denotes 0.001 < *p* ≤ 0.01, and *** denotes 0.0001 < *p* ≤ 0.001.

Three laboratories (LAB_b, LAB_c, and LAB_e) conducted routine detections using the VeriFiler™ Plus kit over multiple quarters. The signal quantities and height distributions of the five dyes in the negative samples are depicted in Figure 2. The results revealed significant differences in negative signal quantities for these labs during the 2021_S3, 2022_S1, and 2021_S4 quarters compared with the other quarters. However, these disparities in signal height distribution did not align perfectly with the signal quantities. In the 2021_S3 quarter, LAB_b exhibited a higher average signal height than in the 2021_S4 and 2022_S1 quarters. LAB_e displayed a markedly lower average signal height for various dyes in the 2020_S2 quarter, in contrast to the other quarters, where the signal height showed no significant differences. LAB_c did not exhibit substantial differences in signal height across the four quarters analyzed. The analysis using the AGCU EX22 kit focused exclusively on LAB_a over 12 quarters (Figure S1). Significant differences in signal quantities were noted among various quarters, particularly in the 2021_S4, 2022_S1, and 2022_S3 quarters, compared with several other quarters in which significant differences in signal quantities were infrequent or relatively weak (0.01 < *p* ≤ 0.05). However, the highest average signal heights were observed in the 2020_S2 and 2020_S3 quarters, with maximum signal heights exceeding 40 RFU. Analysis using the PowerPlex^®^ 21 System kit included results from four laboratories (LAB_c, LAB_d, LAB_e, and LAB_f) (Figure S2). Similar to the previous two kits, varying degrees of differences in negative signal quantities were observed across the different quarters. Notably, these differences seemed to occur randomly, and no regular patterns were observed. Furthermore, the disparity in the results for different dyes varied slightly. Additionally, the differences observed in the signal quantities did not parallel the variations in the signal height distribution. Occasional disparities in height distribution are inevitably poised to exert varying degrees of influence on the exploration of potential information below the AT.

#### 3.1.2. Comparative Analysis of Negative Signal Quantities and Heights across Different Laboratories

**Figure 3:**
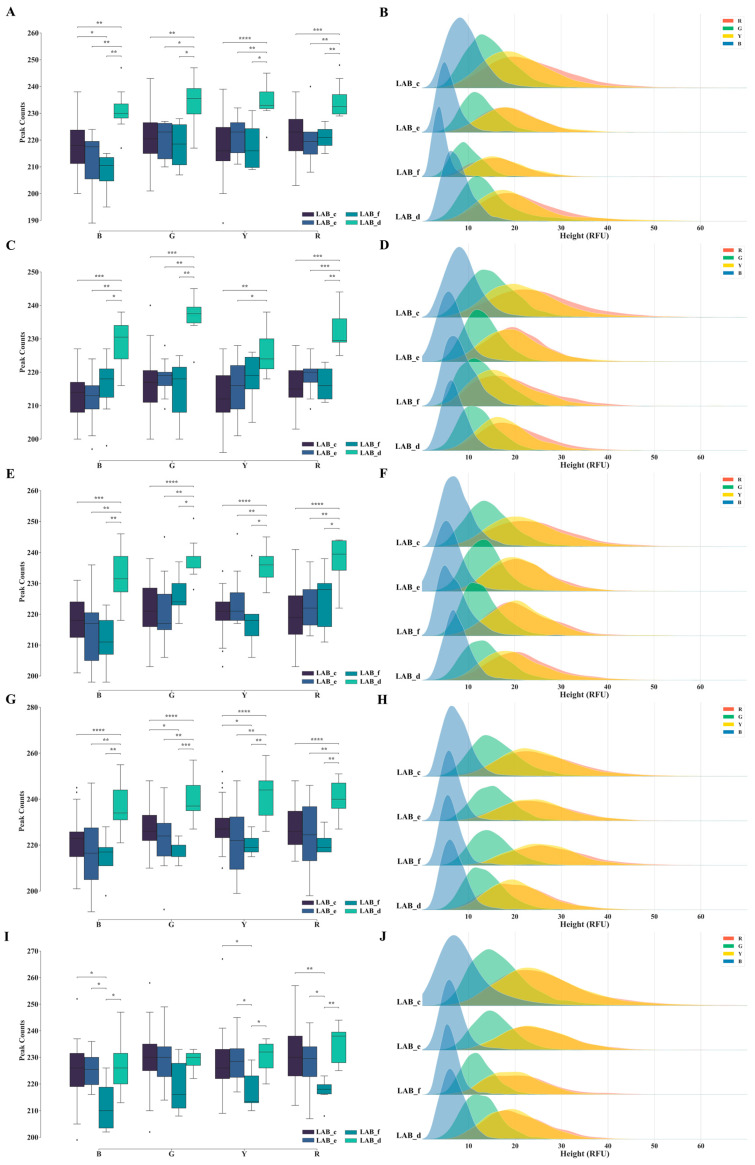
The signal quantities and height distributions in negative samples across four laboratories over five quarters, utilizing the PowerPlex^®^ 21 System kit (LAB_c, d, e, and f). Sub-figures (**A**,**C**,**E**,**G**,**I**) represent the signal quantities for the quarters 2020_S2, 2020_S3, 2020_S4, 2021_S1, and 2021_S2, respectively, while sub-figures (**B**,**D**,**F**,**H**,**J**) illustrate the corresponding signal height distributions. The outliers are represented by diamonds. Statistical significance is indicated by symbols: * denotes 0.01 < *p* ≤ 0.05, ** denotes 0.001 < *p* ≤ 0.01, *** denotes 0.0001 < *p* ≤ 0.001, **** denotes *p* ≤ 0.0001.

A comparison of signal quantities and height distributions in negative samples using the PowerPlex^®^ 21 System kit across the four distinct laboratories is shown in Figure 3. The results from the 2020 (S2, S3, and S4) and 2021 (S1) quarters indicated a significant disparity in the quantity of negative sample signals generated by LAB_d during routine testing, in contrast to other labs. However, there was no notable difference in the height distribution of the negative signals when compared with those of other laboratories, which is consistent with the results of the preceding section. In the 2021_S2 quarter, the difference in the number of negative signals between LAB_d and the other laboratories diminished. During this period, LAB_f produced a considerably lower number of negative signals than the remaining three labs. Notably, the height distribution graph of the negative signals in LAB_c appeared broader than that in the other labs across all five quarters. This suggests that both the average negative signal height and the highest height in LAB_c surpassed those in the other labs. 

Furthermore, the variations in negative sample detection results among labs using the VeriFiler™ Plus kit were relatively minor (Figure S3). Across all three analyzed quarters, the signal quantity of negative samples in LAB_c was significantly lower than that in the other two laboratories, but only in the 2022_S1 quarter. Similar to the findings of the PowerPlex^®^ 21 System kit for detecting negative signals, LAB_c exhibited a higher average negative signal height and the highest signal height compared with those of the other laboratories.

It is essential to acknowledge that different laboratories operate under distinct environmental conditions, and variations in the configuration of their electrophoresis instruments and reagents may lead to potential interpretation discrepancies in the analysis results. Consequently, it is advisable to employ different analysis methods in the process of sample detection and interpretation across these varied laboratory settings.

#### 3.1.3. Analysis of Signal Quantity and Peak Height Distributions in Negative Samples across Different PCR Cycle Numbers

**Figure 4:**
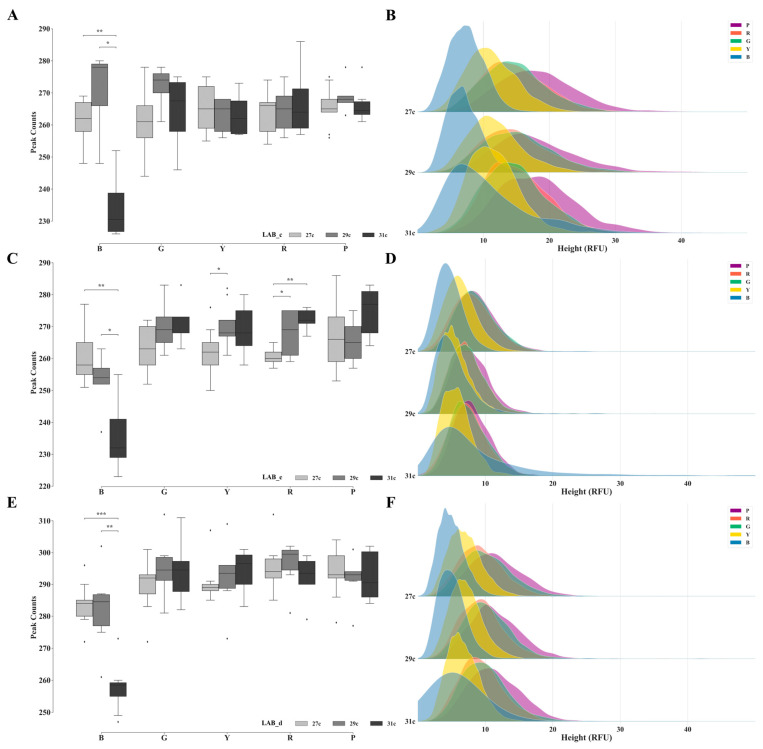
The signal quantities and height distributions in negative samples detected under PCR cycle numbers of 27, 29, and 31, utilizing the VeriFiler™ Plus kit. Sub-figures (**A**,**C**,**E**) display the signal quantities for LAB_c, LAB_e, and LAB_d, respectively, while sub-figures (**B**,**D**,**F**) showcase the corresponding signal height distributions. The outliers are represented by diamonds. Statistical significance is indicated by symbols: * denotes 0.01 < *p* ≤ 0.05, ** denotes 0.001 < *p* ≤ 0.01, *** denotes 0.0001 < *p* ≤ 0.001.

The signal quantity and peak height distributions of the negative samples were compared under different PCR cycle numbers across seven laboratories (Figure 4 and Figure S4). Apart from LAB_a, the results from the remaining six laboratories exhibited a significant decrease in signal quantity for the blue dye after 31 cycles compared with after 27 and 29 cycles. The average and maximum signal heights for the same dye at 31 cycles were notably higher than those observed at the other cycle numbers. No significant differences in the signal quantity and peak height distributions were observed between cycles 27 and 29. Additionally, the results from the seven laboratories did not show a consistent pattern of differences for other dyes. LAB_e and LAB_b demonstrated significant increases and decreases in the signal intensity, respectively, with an increase in the cycle number of the red dye. LAB_g showed the lowest signal intensity after 29 PCR cycles, and no significant differences were noted in the data. The disparities in the signal height distributions were relatively small for the other dyes.

Increasing the number of PCR cycles in routine testing enhances the probability of detecting alleles, primarily affecting negative signals, owing to the simultaneous increase in non-specific amplification products or exogenous DNA. The presence of these products implies the presence of certain levels of external contamination in the laboratory environment or experimental conditions. When laboratories aim to uncover latent genetic information below an AT, the presence of such contamination inevitably influences the interpretation of the results.

### 3.2. Comparison of Methods to Determine AT

#### 3.2.1. ATs Ranged from 1 to 200 RFU

**Figure 5:**
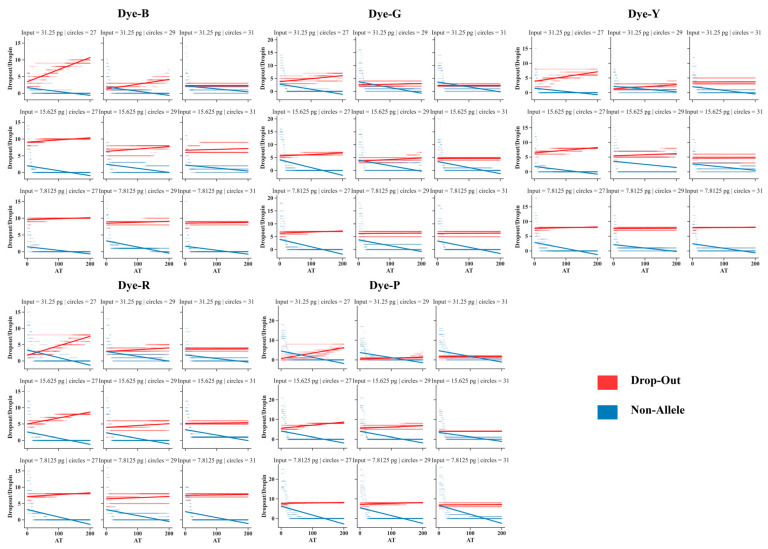
The variation in allele dropouts and non-allele peaks across five dyes for low-template control DNA 9947A detected using the VeriFiler™ Plus kit in LAB_a. The AT was systematically adjusted from 1 to 200 RFU.

When the AT was systematically adjusted from 1 to 200 RFU, the analysis of control DNA 9947A exhibited variations in allelic dropouts and non-allelic peaks across different PCR cycle numbers and template inputs for each dye (Figure 5 and Figures S5–S10). Despite these discrepancies, consistent patterns emerged: allelic dropouts increased with higher ATs, whereas non-allelic peaks decreased under similar conditions. This trend was clear and easy to understand. Specifically, as the number of PCR cycles was increased at the same AT, there was a noticeable reduction in allelic dropouts. Higher ATs led to a gradual flattening of the growth trend of allele dropouts, reaching the lowest achievable level for individual dyes. This stabilization indicated that a certain allele was either completely absent or at an equivalent level to the baseline, rendering detection impossible. For instance, in LAB_a, with a DNA input of 31.25 pg for 9947A, the results for the blue dye indicated that under 27, 29, and 31 PCR cycles, the average numbers of allele dropouts were 4, 2.7, and 2, respectively. Under 31 PCR cycles, the number of allele dropouts remained at the lowest level of 2, despite the increasing AT. Conversely, as the DNA template input decreased under the same AT, allelic dropout increased. Similar to the trend observed with increasing number of PCR cycles, higher ATs led to a gradual flattening of the growth trend of allele dropouts. For example, under 27 PCR cycles and DNA inputs of 31.25 pg, 16.625, and 7.8125 pg, the average numbers of allele dropouts in the blue dye were 4, 9.7, and 9.9, respectively. Furthermore, when the PCR cycle numbers were increased or the DNA template input was reduced, the reduction in the quantity of non-allelic peaks with higher ATs was not significantly pronounced.

Based on these observations, we recommend that when analyzing samples containing low-template DNA, the number of PCR cycles should be increased to enhance the probability of allele detection. However, in samples with extremely low template levels, increasing the number of PCR cycles has minimal impact. It is advisable to raise the AT, ensuring the stability of the dropout quantity at a certain level while minimizing the detection of non-allelic peaks to eliminate interference.

#### 3.2.2. Comparison of Published Methods to Determine ATs

**Figure 6:**
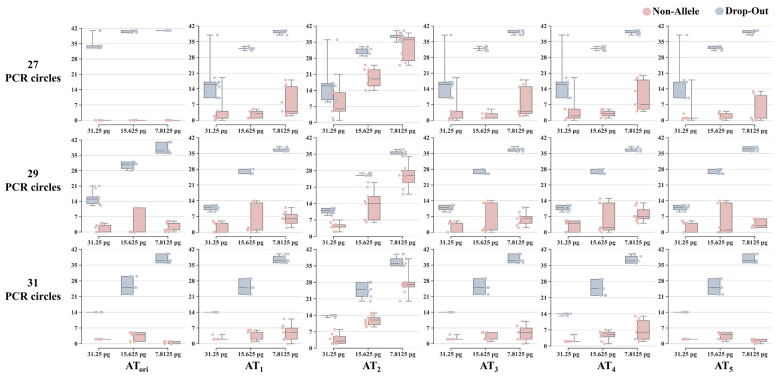
The counts of allele dropouts and non-allele peaks analyzed under six different analytical threshold settings of LAB_a, considering different DNA template inputs and PCR cycle numbers.

A comparison of the results obtained at six different ATs provides valuable insights for optimizing information extraction from electrophoretic data. In this section, LAB_a was used as an example. Table 2 illustrates the six groups of ATs applied to the analysis of the control DNA 9947A. The empirical value of *AT_ori_* was approximately 5.47–15.91 times higher than that of the other groups derived from negative signals. Among these, *AT*_2_ had the smallest threshold value, while *AT*_5_ exhibited the highest threshold value. Figure 6 illustrates the number of allele dropouts and non-allele peaks observed in the six AT settings. When the PCR cycles were set at 27, the number of observed allele dropouts significantly decreased at *AT*_1_–*AT*_5_ compared with that at *AT_ori_*. However, this also led to an increase in the reported non-allele peak counts, especially at lower DNA inputs. *AT*_2_ consistently yielded the highest number of non-allele peaks across different DNA input quantities, surpassing the results obtained in other settings under the same conditions. Increasing the number of PCR cycles to 29 resulted in a less significant reduction in allele dropout, with lower AT values compared to 27 cycles. Under the *AT_ori_* analysis, the count of non-allele peaks exhibited a notable increase. However, for other settings, except for *AT*_2_, substantial increases were observed solely at a DNA input of 15.625 pg, with a decrease noted at 7.8125 pg. At 31 cycles, the allele dropout counts showed no differences among the six AT groups. However, *AT*_2_ exhibited significantly higher non-allele peak counts than those of the other five settings. Among the remaining groups, *AT_ori_* and *AT*_5_ displayed lower non-allele peak counts under the 7.8125 pg DNA input analysis. Similar trends were noted in the results from the other laboratories (Tables S2–S7 and Figures S5–S10). Reanalysis of the profiles using AT calculated from negative signals effectively identified true alleles below the routine AT, thereby reducing dropouts, except in cases with higher PCR cycles. The detection of non-allele peaks increased in some laboratories with an increase in the number of PCR cycles, albeit to varying extents. After adjusting the thresholds, the number of non-allele peaks also increased to varying degrees, possibly because of laboratory environmental conditions, necessitating cautious interpretation of the results. 

**Figure 7:**
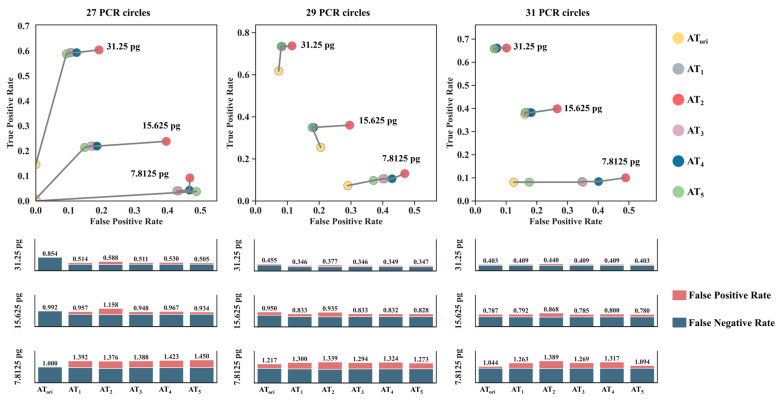
The receiver operating characteristic (ROC) and the total error rate at the six analytical threshold settings of LAB_a, considering different DNA template inputs and PCR cycle numbers.

Figure 7 shows a ROC plot comparing the true-positive and false-positive rates of the results analyzed at six analytical threshold settings. Ideally, the analysis should have a false-positive rate of 0 and a true-positive rate of 1, indicating good performance. Thus, the point closest to the top-left corner represents the optimal method for minimizing Type I and Type II error rates [14]. Figure 7 illustrates the high Type I error rates that occurred when *AT*_2_ was used because of the erroneous labeling of non-allelic signals. In contrast, *AT_ori_* led to low Type I error rates, whereas high thresholds resulted in a high incidence of Type II errors. For DNA template amounts of 31.25 pg or 15.625 pg, irrespective of the number of cycles, *AT*_5_ demonstrated certain advantages, as indicated by the ROC curve. However, when the template amount was extremely low (7.8125 pg), the optimal AT was not discernible from the ROC curve. Considering the total error rate as the sum of false negatives and false positives under all PCR cycles, using *AT_ori_* with a DNA template amount of 7.8125 pg yielded the lowest total error rate, resulting in total error rates of 1.000, 1.217, and 1.044, respectively.

### 3.3. Overview of the NegaProcess Tool

NegaProcess was executed through the command line in the same directory. The input file consists of the “Sizing Table” data generated by analyzing any quantity of negative control samples in Genemapper software. The output comprised two spreadsheet files and a graphic file. The spreadsheets detailed the signal quantity and height distribution in each fluorescence channel for each negative control sample, along with the AT calculated using the five methods. The graphic file visually represents the height distribution of all the negative signals across various fluorescence channels, as shown in Figure 1, providing researchers with a clear visualization of the negative signal distribution. Prior to running the program, users are required to provide five parameters. By typing “NegativeProcess.exe-h”, users can access the meanings of these parameters. The parameters include the path to the “Sizing Table” file of the negative control samples, the number of fluorescence channels used in the STR kit for amplifying these samples (including the internal standards channel), the recommended read region range of this kit, and the maximum x-coordinate for the output graph. This program allows forensic analysts to directly derive thresholds from empirical data, thereby providing a clear and scientifically grounded framework for establishing appropriate analytical thresholds in forensic casework. NegaProcess can be accessed on Google Drive: https://drive.google.com/drive/folders/1BcnO11F5crKli4QBJtw7SKen_CtqaSLr?usp=sharing (accessed on 28 December 2023).

## 4. Discussion

Analysts generally pursue two objectives when detecting and analyzing samples: maximizing obtainable information and minimizing noise. However, there exists a tradeoff between these objectives. The conservative ATs commonly recommended by manufacturers aim to accomplish the latter objective. However, valuable information may exist below the AT, particularly for low-template samples. Indiscriminate reduction of the AT, nonetheless, may result in information that lacks interpretability and fails to provide compelling conclusions in legal contexts.

This study employed two types of detection data: historical data collected from various laboratories, and data obtained from low-template standard samples. The analysis explored the baseline states of negative control samples and assessed the impact of different ATs on both allele and non-allele detection results. The historical data encompassed outcomes from three distinct STR detection kits, with the choice of kit constituting a factor contributing to baseline disparities. The signal height distribution of negative samples from diverse kits exhibited distinctive characteristics, primarily manifested in varying levels of average and maximum peak heights for each dye, owing to different fluorescent labels. Consequently, each kit manufacturer recommends a specific AT.

To examine whether the signal distribution of negative samples changed over time, historical negative samples were categorized into quarters (with each year consisting of four quarters). The results indicated that these differences appeared to occur randomly. Further analysis was conducted to determine if these differences exhibited any seasonal patterns. The findings revealed no correlation with seasonal temperature fluctuations in the quantity or distribution of negative signals across different quarters. Most laboratories operate in controlled environments, maintaining a constant temperature and humidity, thus minimizing the impact of outdoor temperature changes on the experimental process. Random variations may arise from the irregular maintenance of the electrophoresis instruments. Investigating the influence of instrument maintenance changes on experimental results requires dedicated monitoring of the instrument status and thorough record keeping. We advocate that every laboratory should adopt these practices to achieve standardization of experimental conditions. For a more comprehensive interpretation of the analysis results, consider performing repeated tests on the negative control samples after each instrument maintenance session to observe and document the baseline status.

Variations between laboratories are expected, owing to differences in experimental instruments and reagent configurations. In laboratories with stringent control over experimental conditions, the practice of maintaining a lower baseline while adhering to widely accepted ATs may result in the oversight of potential information. This becomes a critical issue, especially in the analysis of challenging materials. In addition, when faced with low-template samples, laboratories typically opt to increase the number of PCR cycles during amplification. Ideally, increasing the number of PCR cycles should exponentially increase the target DNA template quantity and increase the probability of detecting alleles without affecting the negative signals. However, the results of this study indicated that with an increase in PCR cycle numbers, non-specific amplification products or exogenous DNA in the negatives also increased, leading to differences in the distribution of negative signals at different PCR cycle numbers. Specifically, the average and maximum signal heights of the same dye at 31 cycles were significantly higher than those at other cycle numbers. Importantly, these heightened signals did not exceed commonly used thresholds in this study. Designating a profile as contaminated only occurs when these signals exceed the AT, and the quantity surpasses the maximum allowable number of drop-in events (sporadic contamination) [18,19]. Contamination, if present, may be reproducible and can be deduced through different types of negative controls. According to the findings of this study, when opting for an increase in PCR cycle numbers, analysis under conservative thresholds ensures the stability of allele dropout events at a specific level, while concealing non-allele peaks below the AT (Figure 5). Meanwhile, for the analysis of samples with extremely low templates, as defined by quantities below 7.8125 pg in this study, it is imperative to employ conservative ATs to minimize the interference from non-allele peaks.

Currently, various laboratories face a substantial backlog, with certain cases retaining only detection results and no remaining samples for retesting. To reanalyze detection data, there is a pressing need to establish scientific ATs to accurately distinguish allele peaks from noise. This study employed five previously reported methods based on negative signals to calculate the appropriate AT and analyzed the results of low-template standard samples from seven laboratories. Based on the ROC curves, *AT*_5_ consistently exhibited the lowest overall error rate in most cases. This favorable performance can be attributed to *AT*_5_’s calculation, which is grounded in the assumption that negative signals conform to a lognormal distribution. This premise aligns more closely with the observed right-skewed normal distribution of the signal heights for each dye in the various kits. Furthermore, reanalysis of cases showed the significant potential of adjusting the AT based on the *AT*_5_ calculation method to unearth genuine allele information. Notably, in specific cases involving extremely low-template quantities and increased PCR cycle numbers, the original conservative AT exhibited even lower overall error rates, which is consistent with the previously mentioned conclusions.

Moreover, we examined the size and height of non-allele peaks at each locus under each calculated AT. Using the results obtained under *AT*_5_ for LAB_a as an illustration, the occurrence of numerous non-allele peaks was not random; they appeared consistently across samples with different template quantities and PCR cycle numbers (Figure S11). Upon cross-referencing with standard genotyping, regularly occurring non-allele peaks were identified as stutters. Although the analysis process in the GeneMapper ID-X initially filters stutter based on the default stutter ratio of the VeriFiler™ Plus kit, its presence in the current results suggests that the stutter ratio may differ under conditions of low-template DNA, leading to an increased number of non-allele peaks. To address this issue, specialized verification of low-template samples is recommended to eliminate interference from these non-allele peaks during interpretation. Additionally, certain laboratories (such as LAB_c) exhibited a substantial number of spikes and off-ladder (OL) peaks in their analysis results, which contributed to a higher number of outliers in the statistical analysis of non-allele peaks after the AT was lowered. Spike peaks may be caused by external particles, such as dust or dried small aggregates, entering the capillary or gel, or fluctuations in the current [18]. To maintain consistency in sample quantity across laboratories, we chose not to exclude anomalous files. These outliers can be mitigated by increasing sample size. Crucially, when encountering such issues, analysts should promptly review experimental procedures and ensure the proper maintenance of electrophoresis instruments.

This study centers on the analysis of low-template samples, significantly enhancing information availability through the scientific adjustment of the AT. However, it is imperative to acknowledge certain limitations in this research. Without reference genotypes, distinguishing whether peaks above the adjusted AT represent alleles or non-allelic artifacts remains challenging. Forensic researchers have recognized and actively worked toward discerning non-allelic peaks or directly mitigating their impact, particularly with the incorporation of artificial intelligence [10,20,21,22,23,24,25]. Nevertheless, the “black box” issue introduced by sophisticated artificial intelligence algorithms poses a challenge in legal contexts [26]. To meet the criteria of intelligibility and acceptability in court, algorithms addressing such issues must prioritize transparency and readability. Developing a straightforward machine learning algorithm to effectively categorize peaks above the AT for confronting and resolving this persistent challenge is the focal point of our ongoing research.

## 5. Conclusions

In conclusion, this study systematically investigated the baseline signals in electrophoresis results by leveraging data from multiple laboratories. Variability in negative signal distribution was observed across different reagent kits, laboratory conditions, and amplification cycles. Our findings underscore the impact of routine instrument maintenance and reagent changes on baseline levels, providing valuable insights for laboratories conducting forensic DNA analyses. Adjusting the AT according to specific laboratory conditions is crucial for minimizing allele dropout and non-allelic peak detection, ensuring accurate and reliable results. Moreover, a comparative analysis of the five AT calculation methods revealed that barring extreme scenarios of low-template amounts and high PCR cycle numbers, the *AT*_5_ method consistently demonstrated the lowest overall error rate. This suggests that *AT*_5_ is a promising method for enhancing allele detection, particularly in the analysis of challenging historical data. As a practical outcome, we developed a user-friendly program for real-time statistical analysis that facilitates prompt adjustments to the AT based on laboratory-specific conditions. This tool empowers laboratory personnel to conduct efficient and scientifically guided analyses, thereby maximizing information retrieval and ensuring robust forensic DNA analysis. 

In summary, our comprehensive investigation of baseline signals and AT optimization provides valuable insights for forensic DNA analysis. The tailored adjustments recommended in this study, supported by the empirical evidence, offer a practical framework for laboratories to enhance the accuracy and reliability of genetic analysis procedures.

## Figures and Tables

**Table 1:** The overview of the quantity distribution of negative samples collected across six laboratories.

Year	Quarter	LAB_a	LAB_b	LAB_c	LAB_d	LAB_e	LAB_f
2019	S4						17 ^#^
2020	S1	4 *		32 ^+^		11 ^#^	6 ^#^
	S2	25 *		50 ^#^	8 ^#^	8 ^#^/5 ^+^	4 ^#^
	S3	23 *		35 ^#^	6 ^#^	13 ^#^	7 ^#^
	S4	26 *		39 ^#^	10 ^#^	11 ^#^	5 ^#^
2021	S1	29 *		34 ^#^	17 ^#^	10 ^#^	5 ^#^
	S2	23 *		23 ^#^	7 ^#^	12 ^#^	6 ^#^
	S3	26 *	7 ^+^	31 ^+^	12 ^#^	7 ^+^	6 ^#^
	S4	21 *	12 ^+^	17 ^+^	16 ^#^	9 ^+^	4 ^#^
2022	S1	26 *	10 ^+^	56 ^+^		14 ^+^	8 ^#^
	S2	21 *	17 ^+^		25^#^	12 ^+^	
	S3	20 *			8^#^		
	S4	25 *			8^#^		

* AGCU EX22; ^#^ PowerPlex^®^ 21 System; ^+^ VeriFiler™ Plus.

**Table 2:** The analytical threshold values for five dyes applied to analysis in LAB_a, including both the empirical setting and settings calculated using five published methods.

	Dye-B	Dye-G	Dye-Y	Dye-R	Dye-P
*AT_ori_*	175	175	175	175	175
*AT*_1_	16	24	18	22	25
*AT*_2_	11	16	11	15	17
*AT*_3_	17	24	18	23	25
*AT*_4_	16	22	16	21	23
*AT*_5_	22	32	23	29	32

*AT*_1_–*AT*_5_ were calculated based on nine negative controls in low-template DNA experiments (27 PCR circles).

## Data Availability

The data presented in this study are available in article or Supplementary Materials.

## Supplementary Materials

The following supporting information can be downloaded at: https://www.mdpi.com/article/10.3390/genes15010117/s1, Figure S1: The signal quantities and height distributions in negative samples from LAB_a across different quarters, utilizing the AGCU EX22 kit. Figure S2: The signal quantities and height distributions in negative samples from four laboratories across different quarters, utilizing the PowerPlex^®^ 21 System kit. Figure S3: The signal quantities and height distributions in negative samples across three laboratories over five quarters, utilizing the VeriFiler™ Plus kit (LAB_b, c, and e). Figure S4: The signal quantities and height distributions in negative samples detected under PCR cycle numbers of 27, 29, and 31 cycles across three laboratories, utilizing the VeriFiler™ Plus kit. Figures S5–S10: (A) The variation in allele drop-outs and non-allele peaks across five dyes for low-template control DNA 9947A detected using the VeriFiler™ Plus kit in LAB_b–e, g and h. (B) The counts of allele dropouts and non-allele peaks analyzed under six different analytical threshold settings of LAB_b–e, g, and h, considering different DNA template inputs and PCR cycle numbers. (C) The receiver operating characteristic (ROC) and the total error rate at the six analytical threshold settings of LAB_b–e, g, and h, considering different DNA template inputs and PCR cycle numbers. Figure S11: The overview of the size and height of non-allele peaks across five dyes for LAB_a under AT_5_ conditions, considering variations in template quantities and PCR cycle numbers. Table S1: The quantity of signals identified in each dye for three different STR kits. Tables S2–S7: The analytical threshold values for five dyes applied to analysis in LAB_b–e, g, and h, including both the empirical setting and settings calculated using five published methods.
