# Supplementary material for: Optimizing Analytical Thresholds for Low-Template DNA Analysis: Insights from Multi-Laboratory Negative Controls
Source: Genes (Basel). 2024 Jan 18;15(1):117. doi: 10.3390/genes15010117 (PMC10815623; doi:10.3390/genes15010117)
Supplement: Supplementary file 1 [file genes-15-00117-s001.zip › Supplementary File/Supplementary_Figure.pptx]

## Slide 1
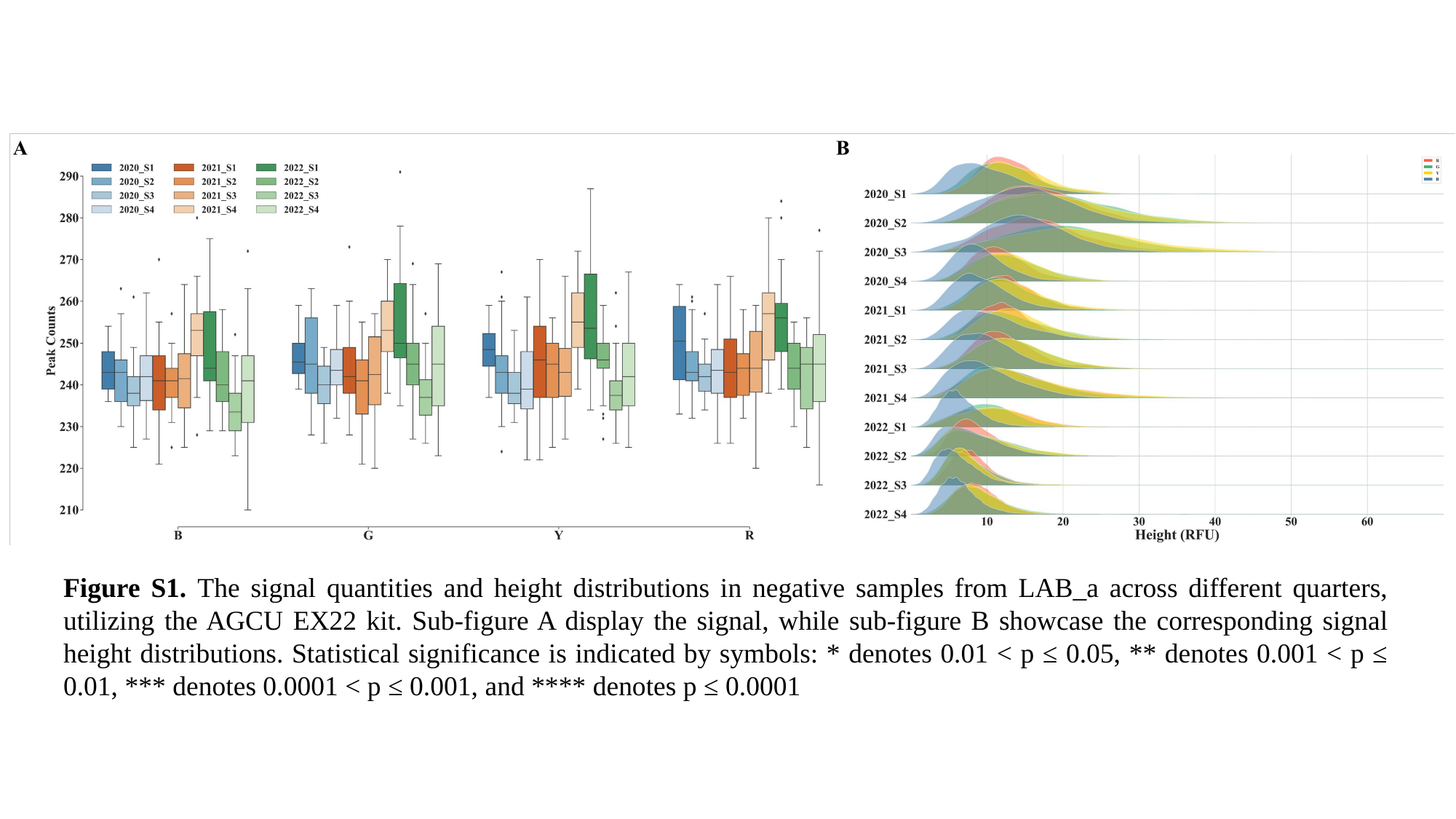

Figure S1. The signal quantities and height distributions in negative samples from LAB_a across different quarters, utilizing the AGCU EX22 kit. Sub-figure A display the signal, while sub-figure B showcase the corresponding signal height distributions. Statistical significance is indicated by symbols: * denotes 0.01 < p ≤ 0.05, ** denotes 0.001 < p ≤ 0.01, *** denotes 0.0001 < p ≤ 0.001, and **** denotes p ≤ 0.0001

## Slide 2
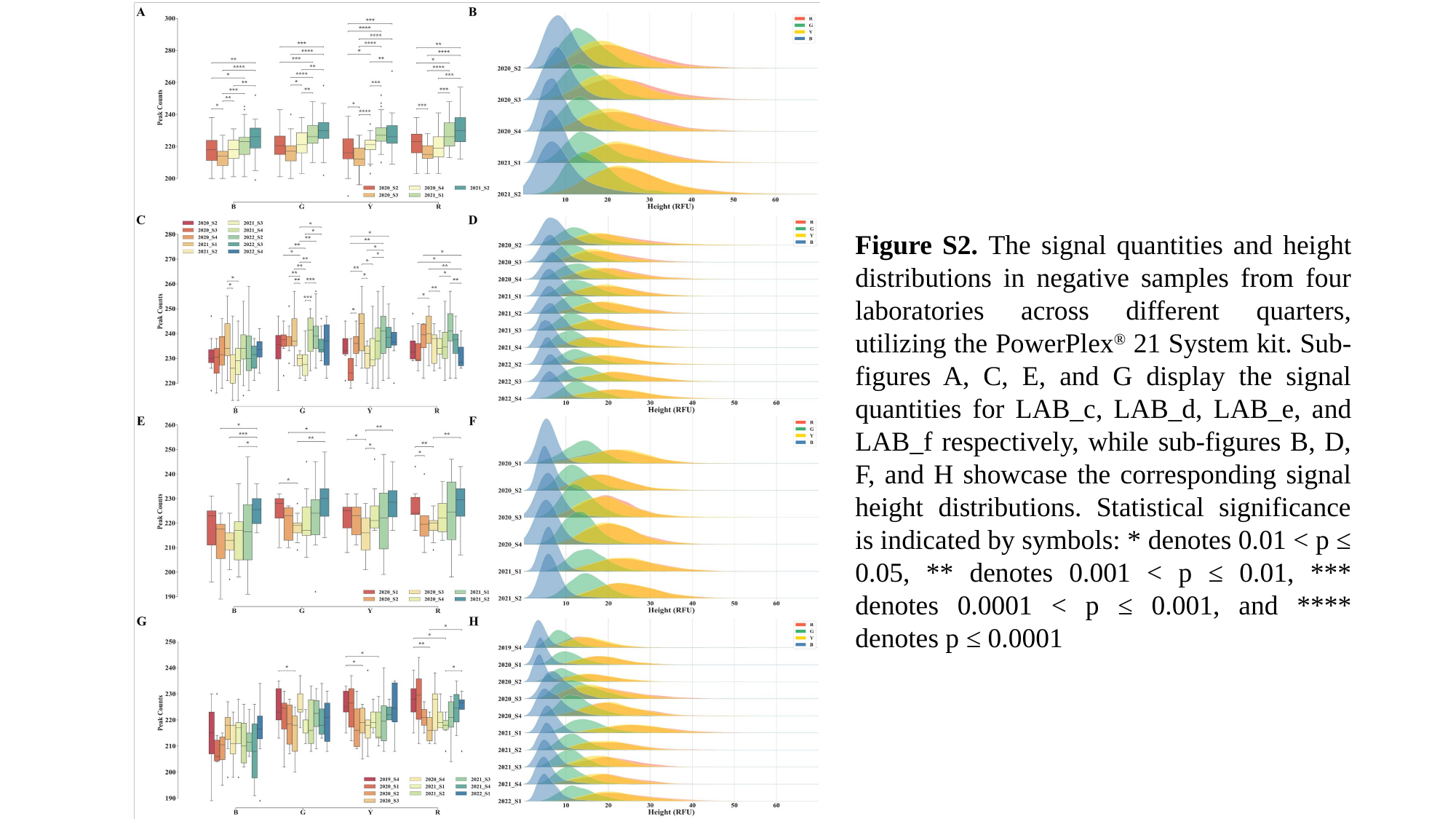

Figure S2. The signal quantities and height distributions in negative samples from four laboratories across different quarters, utilizing the PowerPlex® 21 System kit. Sub-figures A, C, E, and G display the signal quantities for LAB_c, LAB_d, LAB_e, and LAB_f respectively, while sub-figures B, D, F, and H showcase the corresponding signal height distributions. Statistical significance is indicated by symbols: * denotes 0.01 < p ≤ 0.05, ** denotes 0.001 < p ≤ 0.01, *** denotes 0.0001 < p ≤ 0.001, and **** denotes p ≤ 0.0001

## Slide 3
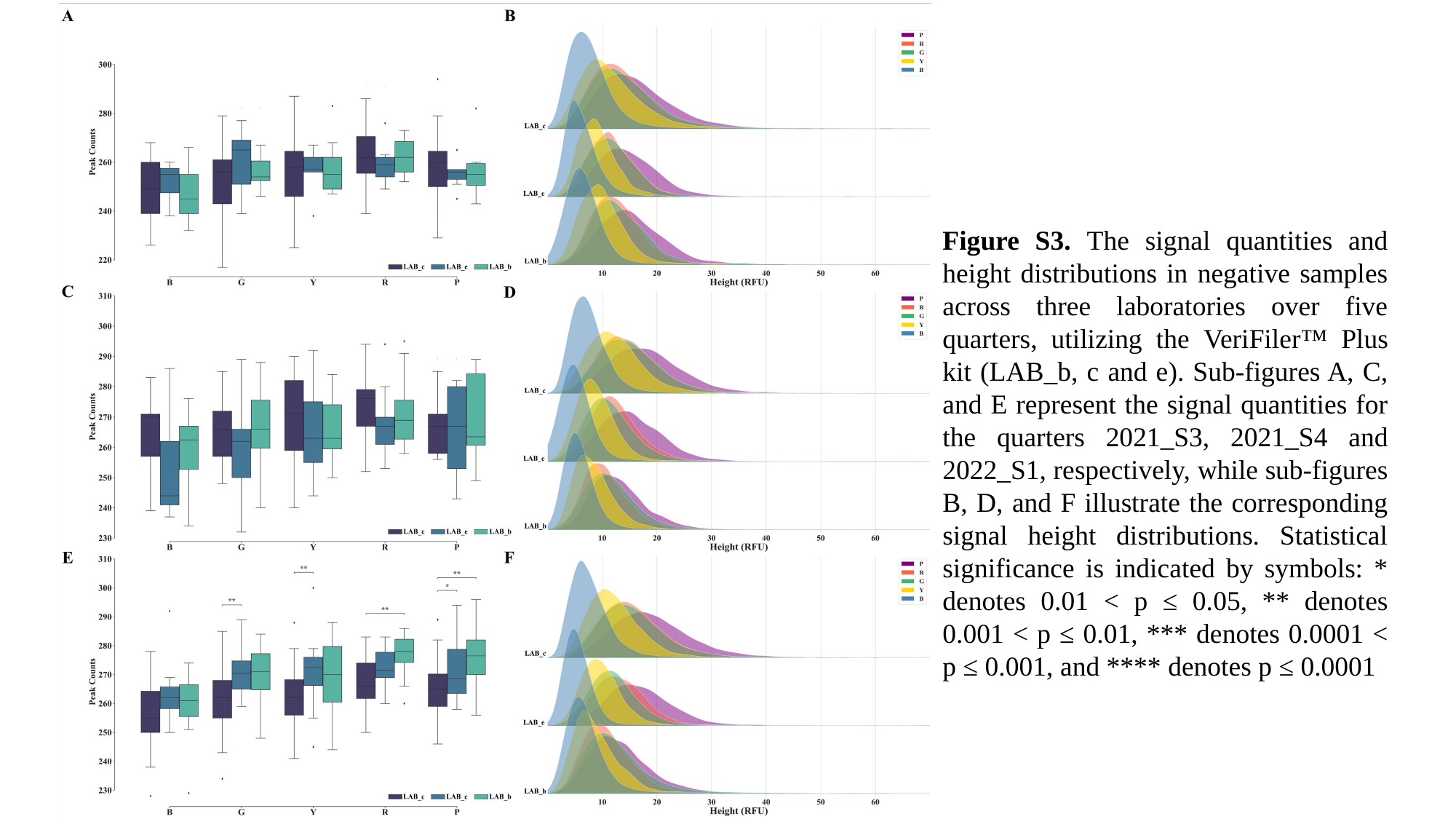

Figure S3. The signal quantities and height distributions in negative samples across three laboratories over five quarters, utilizing the VeriFiler™ Plus kit (LAB_b, c and e). Sub-figures A, C, and E represent the signal quantities for the quarters 2021_S3, 2021_S4 and 2022_S1, respectively, while sub-figures B, D, and F illustrate the corresponding signal height distributions. Statistical significance is indicated by symbols: * denotes 0.01 < p ≤ 0.05, ** denotes 0.001 < p ≤ 0.01, *** denotes 0.0001 < p ≤ 0.001, and **** denotes p ≤ 0.0001

## Slide 4
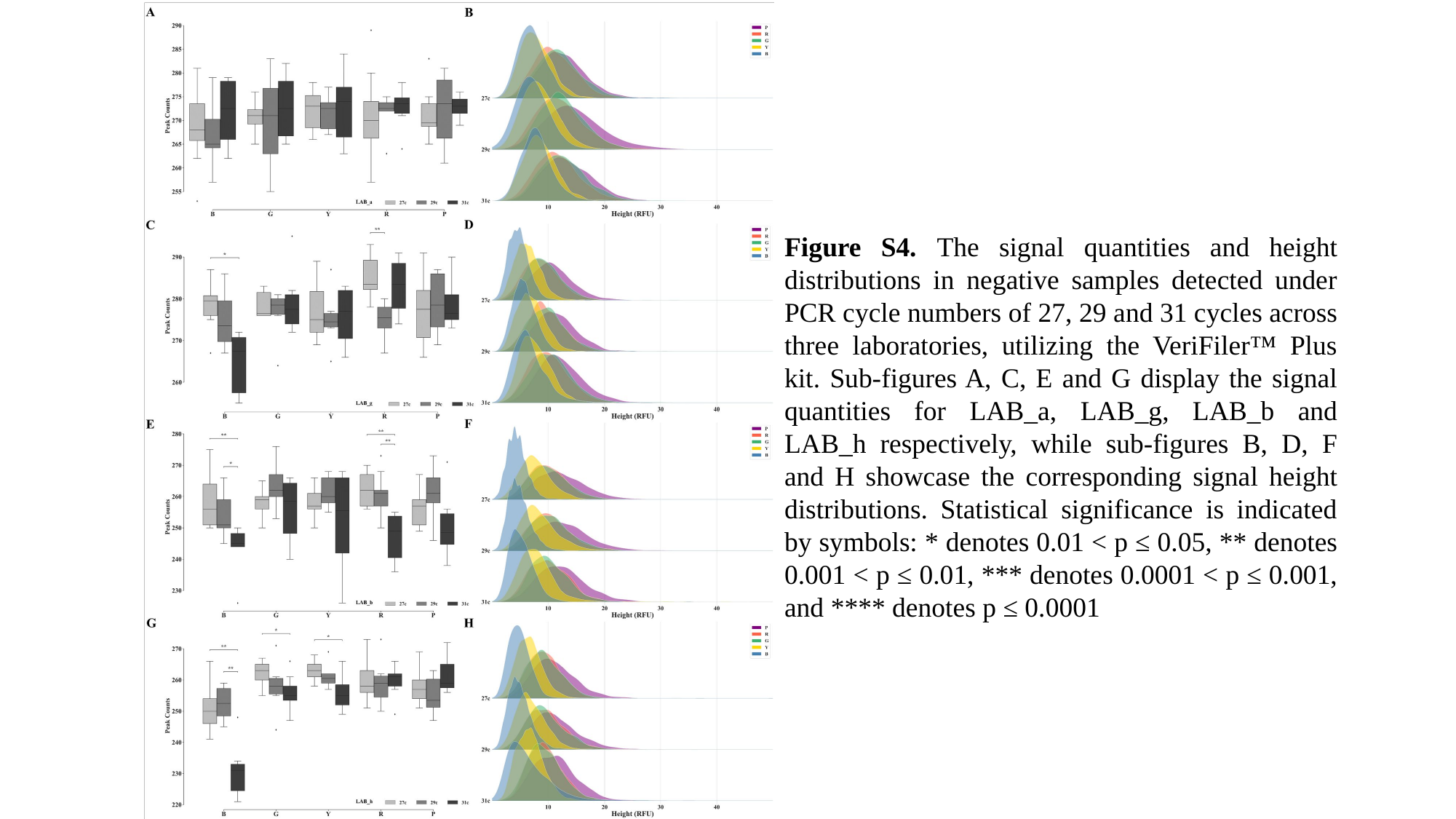

Figure S4. The signal quantities and height distributions in negative samples detected under PCR cycle numbers of 27, 29 and 31 cycles across three laboratories, utilizing the VeriFiler™ Plus kit. Sub-figures A, C, E and G display the signal quantities for LAB_a, LAB_g, LAB_b and LAB_h respectively, while sub-figures B, D, F and H showcase the corresponding signal height distributions. Statistical significance is indicated by symbols: * denotes 0.01 < p ≤ 0.05, ** denotes 0.001 < p ≤ 0.01, *** denotes 0.0001 < p ≤ 0.001, and **** denotes p ≤ 0.0001

## Slide 5
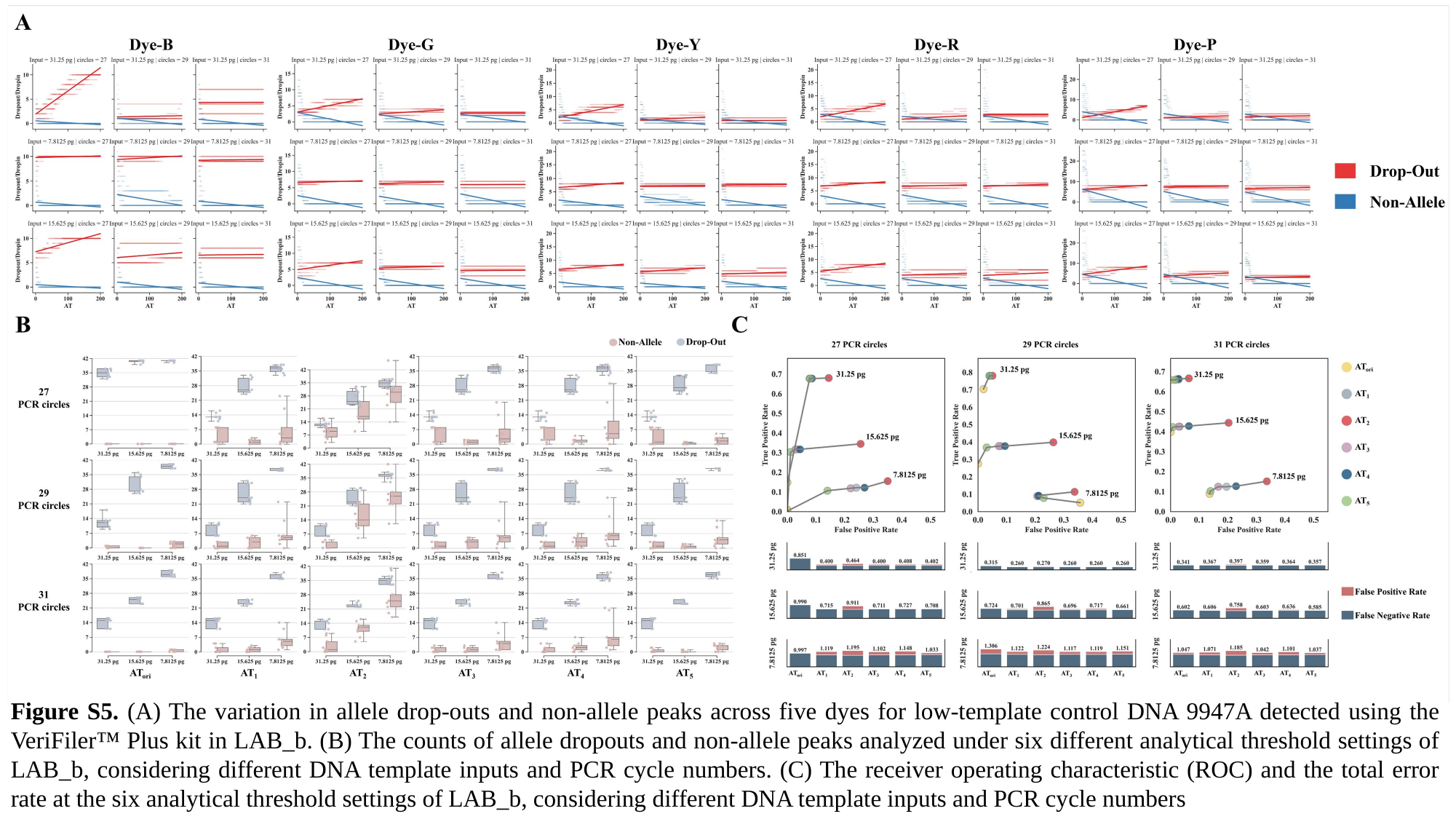

Figure S5. (A) The variation in allele drop-outs and non-allele peaks across five dyes for low-template control DNA 9947A detected using the VeriFiler™ Plus kit in LAB_b. (B) The counts of allele dropouts and non-allele peaks analyzed under six different analytical threshold settings of LAB_b, considering different DNA template inputs and PCR cycle numbers. (C) The receiver operating characteristic (ROC) and the total error rate at the six analytical threshold settings of LAB_b, considering different DNA template inputs and PCR cycle numbers

## Slide 6
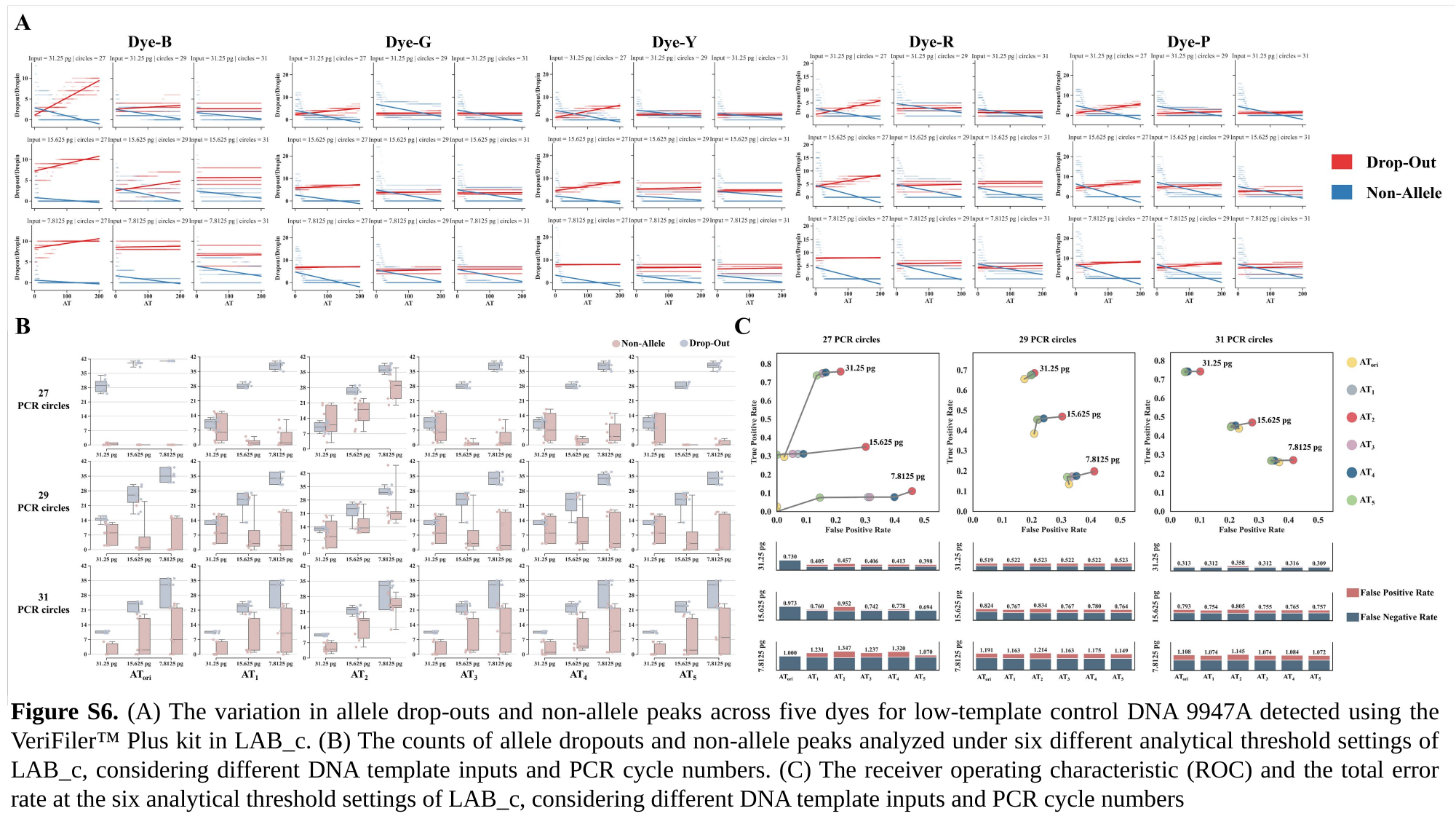

Figure S6. (A) The variation in allele drop-outs and non-allele peaks across five dyes for low-template control DNA 9947A detected using the VeriFiler™ Plus kit in LAB_c. (B) The counts of allele dropouts and non-allele peaks analyzed under six different analytical threshold settings of LAB_c, considering different DNA template inputs and PCR cycle numbers. (C) The receiver operating characteristic (ROC) and the total error rate at the six analytical threshold settings of LAB_c, considering different DNA template inputs and PCR cycle numbers

## Slide 7
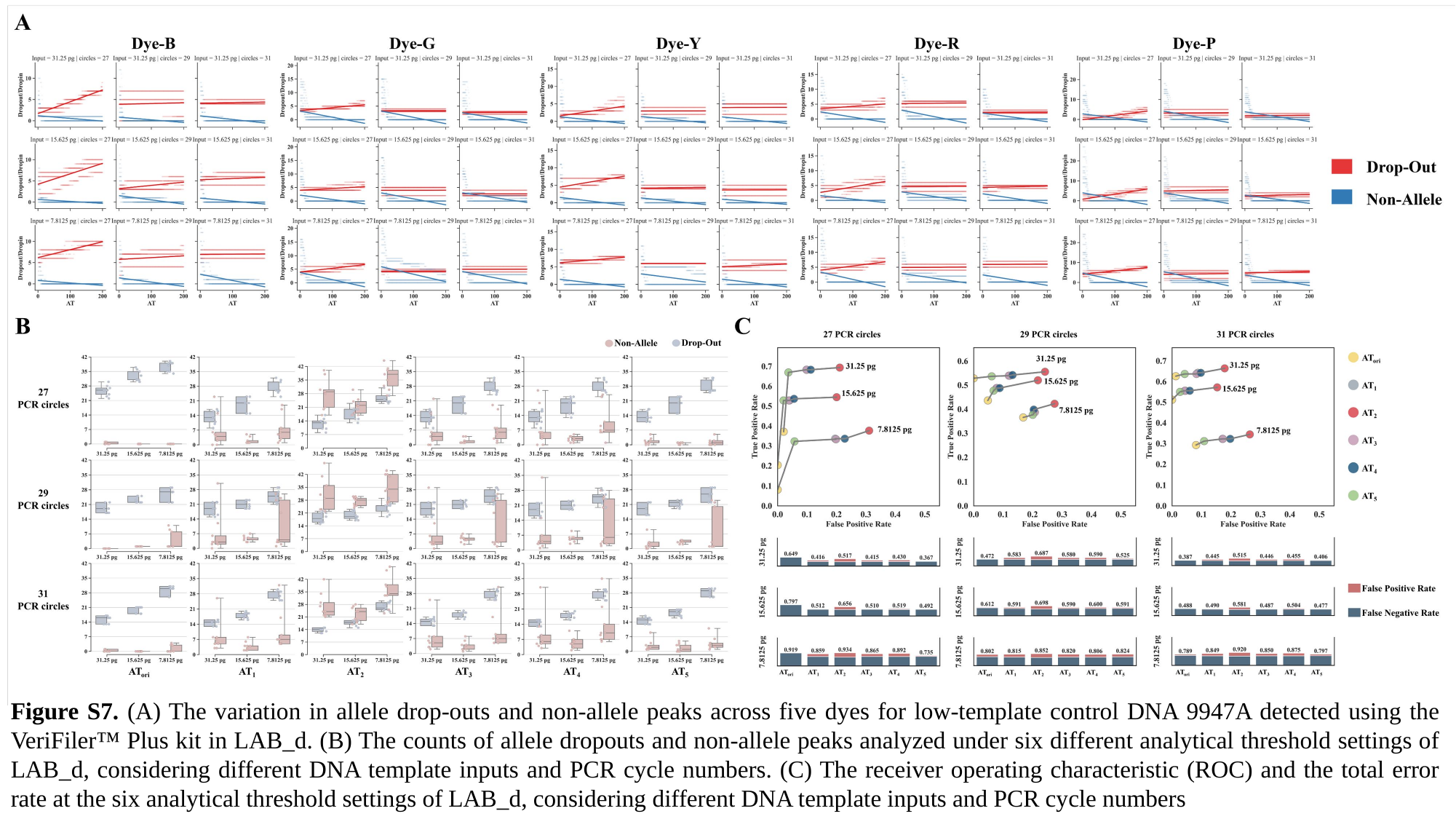

Figure S7. (A) The variation in allele drop-outs and non-allele peaks across five dyes for low-template control DNA 9947A detected using the VeriFiler™ Plus kit in LAB_d. (B) The counts of allele dropouts and non-allele peaks analyzed under six different analytical threshold settings of LAB_d, considering different DNA template inputs and PCR cycle numbers. (C) The receiver operating characteristic (ROC) and the total error rate at the six analytical threshold settings of LAB_d, considering different DNA template inputs and PCR cycle numbers

## Slide 8
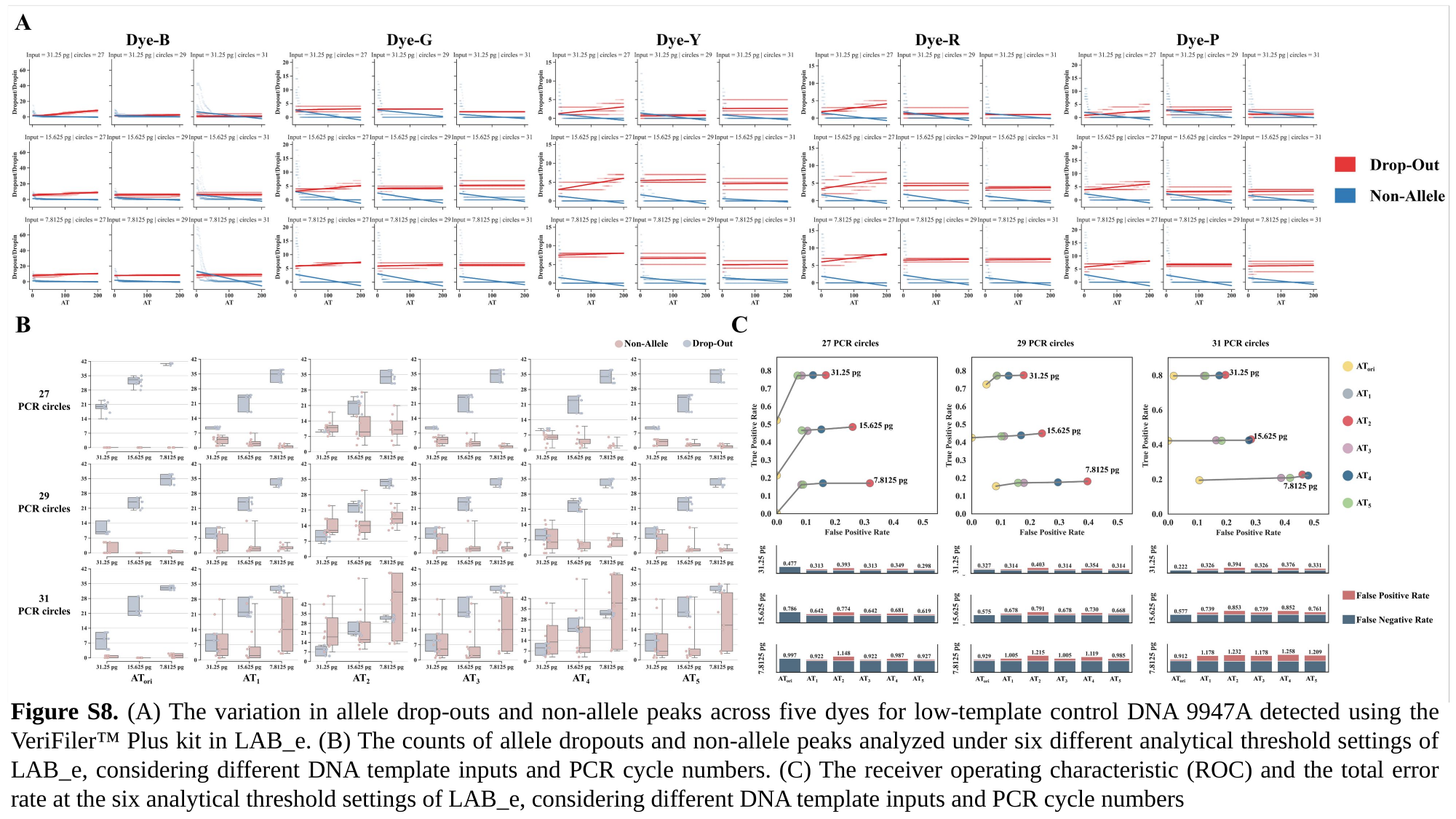

Figure S8. (A) The variation in allele drop-outs and non-allele peaks across five dyes for low-template control DNA 9947A detected using the VeriFiler™ Plus kit in LAB_e. (B) The counts of allele dropouts and non-allele peaks analyzed under six different analytical threshold settings of LAB_e, considering different DNA template inputs and PCR cycle numbers. (C) The receiver operating characteristic (ROC) and the total error rate at the six analytical threshold settings of LAB_e, considering different DNA template inputs and PCR cycle numbers

## Slide 9
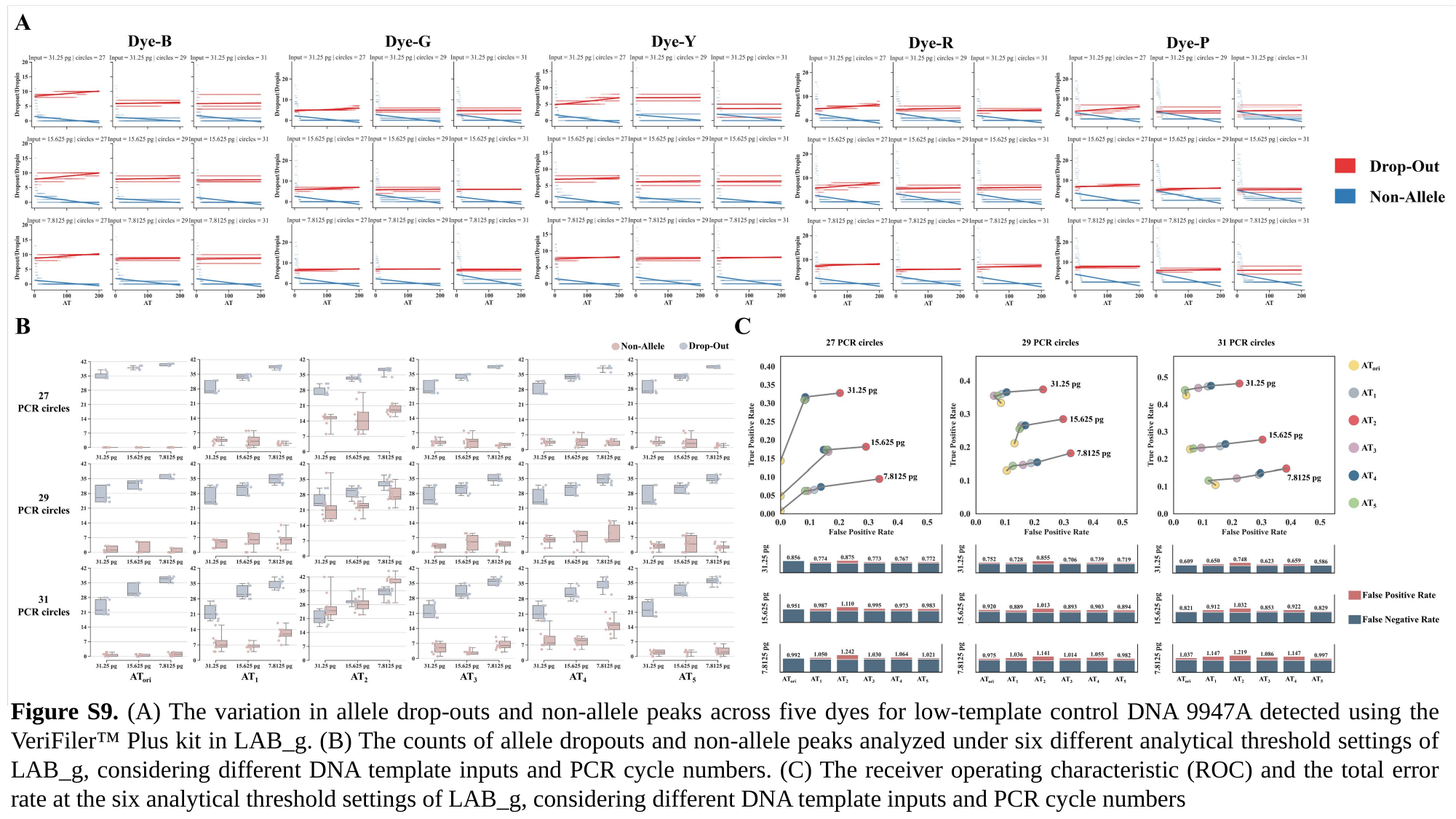

Figure S9. (A) The variation in allele drop-outs and non-allele peaks across five dyes for low-template control DNA 9947A detected using the VeriFiler™ Plus kit in LAB_g. (B) The counts of allele dropouts and non-allele peaks analyzed under six different analytical threshold settings of LAB_g, considering different DNA template inputs and PCR cycle numbers. (C) The receiver operating characteristic (ROC) and the total error rate at the six analytical threshold settings of LAB_g, considering different DNA template inputs and PCR cycle numbers

## Slide 10
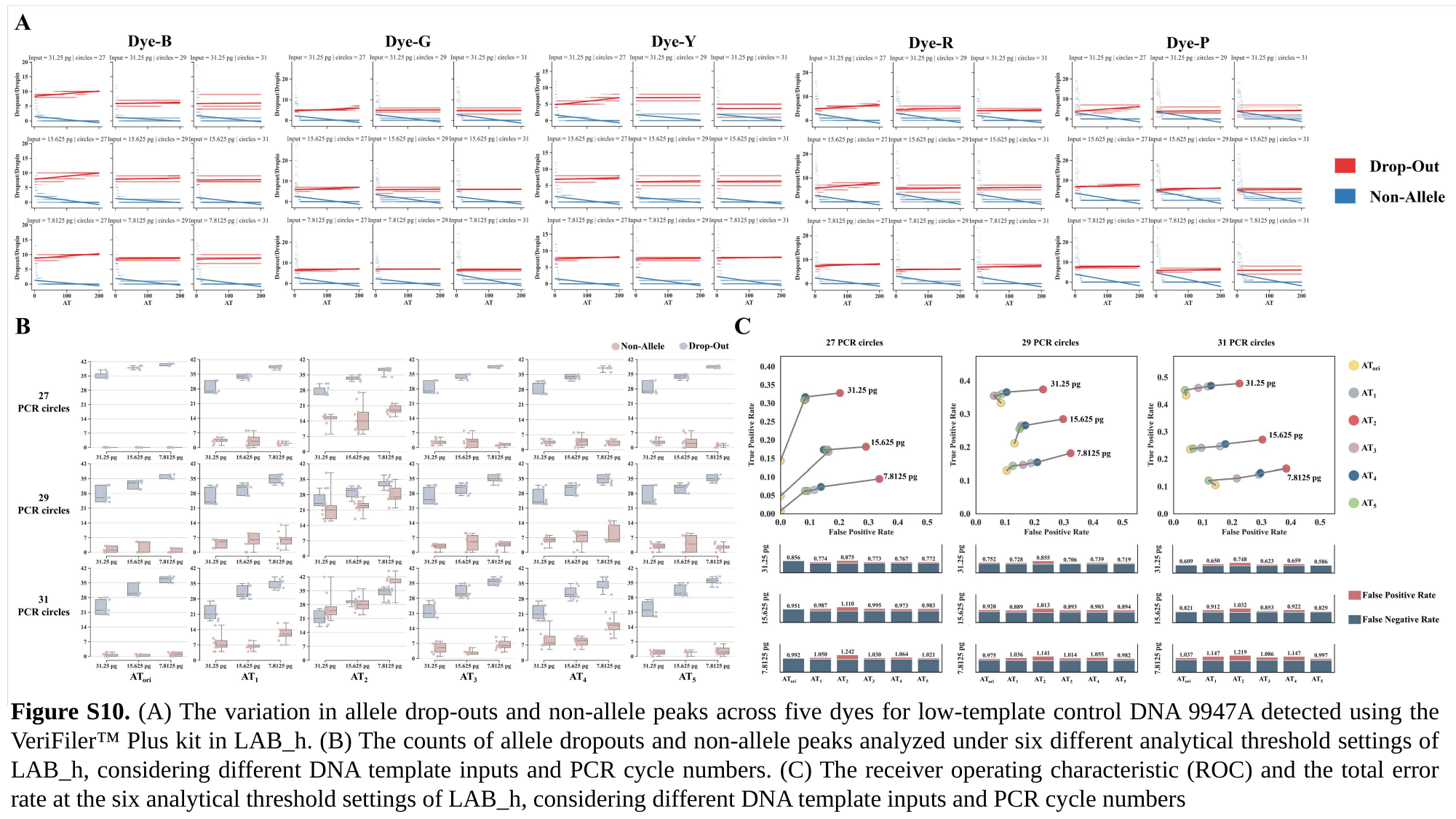

Figure S10. (A) The variation in allele drop-outs and non-allele peaks across five dyes for low-template control DNA 9947A detected using the VeriFiler™ Plus kit in LAB_h. (B) The counts of allele dropouts and non-allele peaks analyzed under six different analytical threshold settings of LAB_h, considering different DNA template inputs and PCR cycle numbers. (C) The receiver operating characteristic (ROC) and the total error rate at the six analytical threshold settings of LAB_h, considering different DNA template inputs and PCR cycle numbers

## Slide 11
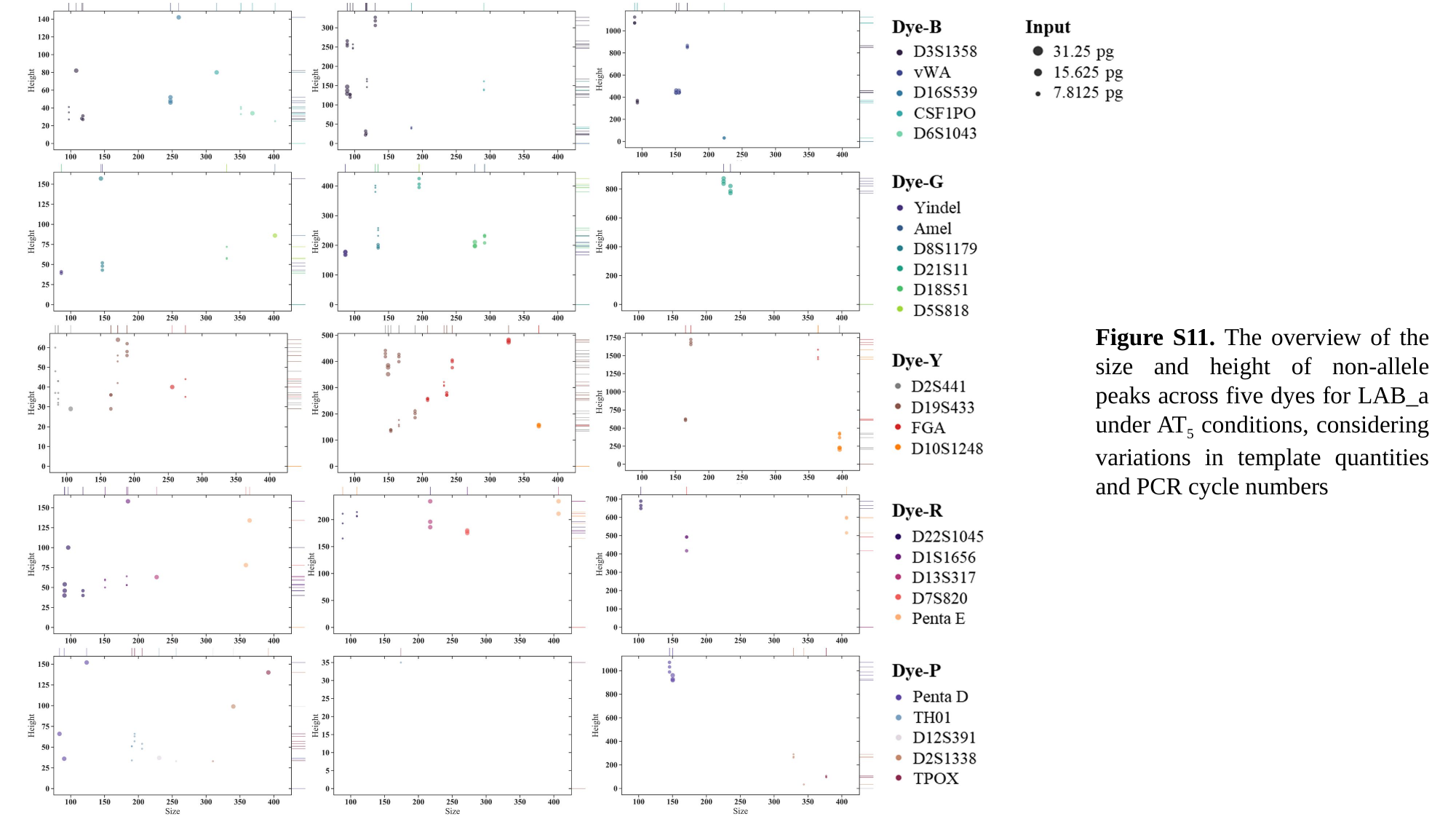

Figure S11. The overview of the size and height of non-allele peaks across five dyes for LAB_a under AT5 conditions, considering variations in template quantities and PCR cycle numbers
